# Bis(*O*-*n*-butyl dithio­carbonato-κ^2^ *S*,*S*′)bis­(pyridine-κ*N*)manganese(II)

**DOI:** 10.1107/S1600536811026523

**Published:** 2011-07-09

**Authors:** Naveed Alam, Muhammad Ali Ehsan, Matthias Zeller, Muhammad Mazhar, Zainudin Arifin

**Affiliations:** a Department of Chemistry, Quaid-I-Azam University, Islamabad 45320, Pakistan; b Department of Chemistry, University of Malaya, 50603 Kuala Lumpur, Malaysia; c Department of Chemistry, Youngstown State University, 1 University Plaza, Youngstown, Ohio 44555, USA

## Abstract

The structure of the title manganese complex, [Mn(C_5_H_9_OS_2_)_2_(C_5_H_5_N)_2_] or [Mn(S_2_CO-*n*-*Bu*)_2_(C_5_H_5_N)_2_], consists of discrete monomeric entities with Mn^2+^ ions located on centres of inversion. The metal atom is coordinated by a six-coordinate *trans*-N_2_S_4_ donor set with the pyridyl N atoms located in the apical positions. The observed slight deviations from octa­hedral geometry are caused by the bite angle of the bidentate κ^2^-S_2_CO-*n*-*Bu* ligands [69.48 (1)°]. The O(CH_2_)_3_(CH_3_) chains of the *O*-*n*-butyl dithio­carbonate units are disordered over two sets of sites with an occupancy ratio of 0.589 (2):0.411 (2).

## Related literature

For general background to the title complex, see: Alam *et al.* (2008 ▶); Tahir *et al.* (2010 ▶); Klevtsova & Glinskaya (1997 ▶); Câmpian *et al.* (2010 ▶); Kirichenko *et al.* (1994 ▶).
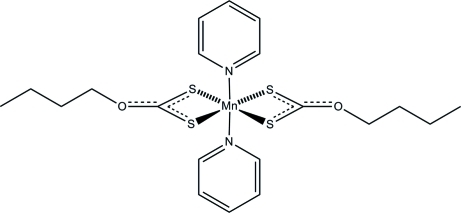


## Experimental

### 

#### Crystal data

[Mn(C_5_H_9_OS_2_)_2_(C_5_H_5_N)_2_]
*M* *_r_* = 511.66
Monoclinic, 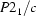

*a* = 10.9189 (17) Å
*b* = 6.0853 (9) Å
*c* = 17.650 (3) Å
β = 97.536 (3)°
*V* = 1162.6 (3) Å^3^
*Z* = 2
Mo *K*α radiation
μ = 0.95 mm^−1^
*T* = 100 K
0.50 × 0.37 × 0.26 mm

#### Data collection

Bruker SMART APEX CCD diffractometer
Absorption correction: multi-scan (*SADABS*; Bruker, 2003 ▶) *T*_min_ = 0.630, *T*_max_ = 0.782
11354 measured reflections
2871 independent reflections
2797 reflections with *I* > 2σ(*I*)
*R*_int_ = 0.028

#### Refinement

*R*[*F* ^2^ > 2σ(*F* ^2^)] = 0.025
*wR*(*F* ^2^) = 0.064
*S* = 1.12
2871 reflections
151 parameters
1 restraint
H-atom parameters constrained
Δρ_max_ = 0.34 e Å^−3^
Δρ_min_ = −0.50 e Å^−3^

### 

Data collection: *SMART* (Bruker, 2002 ▶); cell refinement: *SAINT-Plus* (Bruker, 2002 ▶); data reduction: *SAINT-Plus*; program(s) used to solve structure: *SHELXTL* (Sheldrick, 2008 ▶); program(s) used to refine structure: *SHELXTL*; molecular graphics: *SHELXTL*; software used to prepare material for publication: *publCIF* (Westrip, 2010 ▶).

## Supplementary Material

Crystal structure: contains datablock(s) I, global. DOI: 10.1107/S1600536811026523/rk2277sup1.cif

Structure factors: contains datablock(s) I. DOI: 10.1107/S1600536811026523/rk2277Isup2.hkl

Additional supplementary materials:  crystallographic information; 3D view; checkCIF report

## Figures and Tables

**Fig. 1.**
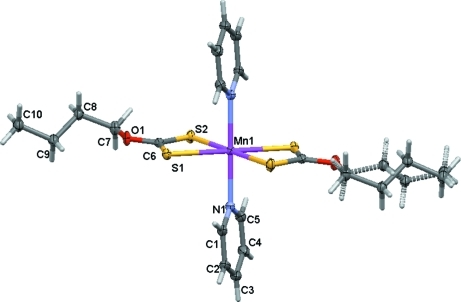
Perspective view of the monomeric unit with the atomic numbering scheme. Displacement ellipsoids are drawn at with 50% probability level. The minor disordered alkyl chain is shown for one half of the molecule. Hydrogen atom lables, labels of less than 50% occupied atoms and of symmetry created atoms are omitted for clarity.

**Fig. 2.**
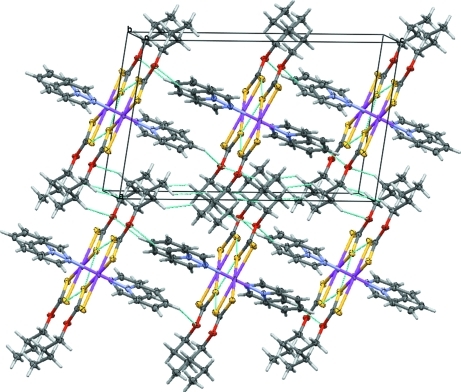
Packing plot, view down the b axis (displacement ellipsoids for non-H atoms). Less than 50% occupied atoms are omitted for clarity. Blue dashed lines represent close contacts (less than the sum of the van der Waals radii of the respective atoms) such as C—H···O and S···S contacts.

## supplementary crystallographic information

### Comment

As a part of our ongoing studies on the development of single source precursors for the fabrication of pure manganese sulfide thin films through aerosol-assisted chemical vapour deposition (AACVD) (Alam *et al.*, 2008; Tahir *et al.*, 2010) the monomeric title complex [Mn(S_2_CO-*n*-*Bu*)_2_^.^(C_5_H_5_N)_2_] was synthesized and its crystal structure, one of just four manganese dithiocarbonates (Klevtsova & Glinskaya, 1997; Câmpian *et al.*, 2010; Kirichenko *et al.*, 1994), is reported here.

The structure of the manganese compound consists of centrosymmetric monomeric entities. Fig. 1 shows a perspective view of the monomeric unit with the atomic numbering scheme. The Mn(II) atom is in a distorted octahedral environment surrounded by two chelating xanthate ligands and two pyridines ligands. All managanese dithiocarbonato compounds structurally described so far are also octahedral complexes with an N_2_S_4_ donor set (Klevtsova & Glinskaya, 1997; Câmpian *et al.*, 2010; Kirichenko *et al.*, 1994), but the other four such compounds are all bipyridine derivative complexes and the title compound is the only one in which the two none-sulfur donor atoms occupy the apical sites. The four sulfur atoms and the manganese atom are almost coplanar. The bond angles around the manganese atom are in the range of 69.48 (1)° to 180°. The Mn—S bond lengths involving the xanthate ligands range are 2.5862 (4) and 2.6556 (5)Å and are in good agreement with those reported for other analogous Mn-dithiocarbonato complexes. The variation of the Mn—S bond distances in the complex of *ca* 0.07Å is not very pronounced and the bidentate κ^2^-S_2_CO-*n*-*Bu* ligands may thus be considered to be chelating in a symmetric (isobidentate) mode. The resulting N_2_S_4_ donor set defines an approximately octahedral geometry with distortions arising from the steric constraints imposed by the restricted bite distances of the chelating xanthate ligands. The two S atoms forming the longer Mn—S bonds are approximately *trans* to each other. The short value of 1.333 (8)Å for the C6—O1 bond lengths is consistent with a significant contribution of the resonance form of the xanthate anion that features a formal C═O bond and negative charges on each of the S atoms. The two pyridine rings are coplanar and almost perfectly perpendicular to the O1/S1/S2/O1^i^/S1^i^/S2^i^ plane. Symmetry code: (i) -*x*+1, -*y*+1, -*z*+2.

Packing of the title compound is facilitated mostly by shape recognition through van der Waals forces. A small number of C—H···O interactions (originating from the alkyl and aromatic C—H groups) can be observed (Fig. 2), and close contacts are present between sulfur atoms of neighboring complexes. These close contacts weakly connect the MnS_4_ units of the complexes along the direction of the *b*-axis to form infinite (MnS_4_)_n_ chains as shown in Fig. 2.

### Experimental

Sodium hydroxide (3.99 g, 0.1 mol) was dissolved in 250 ml *n*-butanol placed in a 500 ml oven dried round bottom flask fitted with reflux condenser, magnetic stirrer and vaccum line. Carbon disulfide (7.6 ml, 0.1 mol) was added dropwise to the saturated solution of sodium hydroxide over a period of 90 minutes. After stirring for one hour a clear yellow solution was formed. Mn(NO_3_)_2_^.^3H_2_O (11.64 g, 0.05 mole) was added directly into the reaction flask. The contents were stirred to dissolve the salt completely. About 30 ml of pyridine were added to give a light yellow solution and stirring was continued for another hour. Any insoluble matter was removed by filtration and slow evaporation of the reaction mixture at room temperature yielded 70% of the title compound in the form of yellow crystals. M.p. = 368 K. Elemental analyses: Found (Calc.) for: C 46.02 (46.99); H 5.33 (5.51); N 4.58 (5.47).

### Refinement

Reflections 1 0 0 and 0 0 2 were partially obstructed by the beam stop and were omitted from the refinement. The O(CH_2_)_3_(CH_3_) chain of the *O*-*n*-butyldithiocarbonato group is disordered over two positions with an occupancy ratio of 0.589 (2) to 0.411 (2). The C—O bond distance was restrained to be the same within a standard deviation of 0.02, and the ADPs of equivalent atoms were set to be identical. Hydrogen atoms were placed in calculated positions with C—H distances of 0.95, 0.99 and 0.99Å for aromatic, methyl and methylene H atoms, respectively, and were refined with an isotropic displacement parameter *U*_iso_ of 1.5 (methyl) or 1.2 times (aromatic) that of *U*_eq_ of the adjacent carbon atom. Methyl H atoms were allowed to rotate around the C—C bond axis at a fixed angle to best fit with the experimental electron density.

### Crystal data

**Table:**

[Mn(C_5_H_9_OS_2_)_2_(C_5_H_5_N)_2_]	*F*(000) = 534
*M**_r_* = 511.66	*D*_x_ = 1.462 Mg m^−^^3^
Monoclinic, *P*2_1_/*c*	Melting point: 368 K
Hall symbol: -P 2ybc	Mo *K*α radiation, λ = 0.71073 Å
*a* = 10.9189 (17) Å	Cell parameters from 6031 reflections
*b* = 6.0853 (9) Å	θ = 2.8–30.7°
*c* = 17.650 (3) Å	µ = 0.95 mm^−^^1^
β = 97.536 (3)°	*T* = 100 K
*V* = 1162.6 (3) Å^3^	Block, yellow
*Z* = 2	0.50 × 0.37 × 0.26 mm

### Data collection

**Table:**

Bruker SMART APEX CCD diffractometer	2871 independent reflections
Radiation source: fine-focus sealed tube	2797 reflections with *I* > 2σ(*I*)
graphite	*R*_int_ = 0.028
ω scans	θ_max_ = 28.3°, θ_min_ = 2.8°
Absorption correction: multi-scan (*SADABS*; Bruker, 2003)	*h* = −14→14
*T*_min_ = 0.630, *T*_max_ = 0.782	*k* = −8→8
11354 measured reflections	*l* = −23→23

### Refinement

**Table:**

Refinement on *F*^2^	Primary atom site location: structure-invariant direct methods
Least-squares matrix: full	Secondary atom site location: difference Fourier map
*R*[*F*^2^ > 2σ(*F*^2^)] = 0.025	Hydrogen site location: inferred from neighbouring sites
*wR*(*F*^2^) = 0.064	H-atom parameters constrained
*S* = 1.12	*w* = 1/[σ^2^(*F*_o_^2^) + (0.0232*P*)^2^ + 0.6054*P*] where *P* = (*F*_o_^2^ + 2*F*_c_^2^)/3
2871 reflections	(Δ/σ)_max_ = 0.001
151 parameters	Δρ_max_ = 0.34 e Å^−^^3^
1 restraint	Δρ_min_ = −0.50 e Å^−^^3^

### Special details

**Table:**

Geometry. All s.u.'s (except the s.u. in the dihedral angle between two l.s. planes) are estimated using the full covariance matrix. The cell s.u.'s are taken into account individually in the estimation of s.u.'s in distances, angles and torsion angles; correlations between s.u.'s in cell parameters are only used when they are defined by crystal symmetry. An approximate (isotropic) treatment of cell s.u.'s is used for estimating s.u.'s involving l.s. planes.
Refinement. Refinement of *F*^2^ against ALL reflections. The weighted *R*-factor *wR* and goodness of fit *S* are based on *F*^2^, conventional *R*-factors *R* are based on *F*, with *F* set to zero for negative *F*^2^. The threshold expression of *F*^2^ > σ(*F*^2^) is used only for calculating *R*-factors(gt) *etc*. and is not relevant to the choice of reflections for refinement. *R*-factors based on *F*^2^ are statistically about twice as large as those based on *F*, and *R*-factors based on ALL data will be even larger.

### Fractional atomic coordinates and isotropic or equivalent isotropic displacement parameters (Å^2^)

**Table:**

	*x*	*y*	*z*	*U*_iso_*/*U*_eq_	Occ. (<1)
C1	0.57264 (12)	0.6534 (2)	1.17119 (7)	0.0219 (3)	
H1A	0.5302	0.7817	1.1516	0.026*	
C2	0.61621 (13)	0.6439 (2)	1.24896 (7)	0.0261 (3)	
H2A	0.6034	0.7634	1.2817	0.031*	
C3	0.67842 (14)	0.4574 (3)	1.27770 (7)	0.0282 (3)	
H3A	0.7083	0.4460	1.3306	0.034*	
C4	0.69638 (13)	0.2874 (2)	1.22795 (8)	0.0271 (3)	
H4A	0.7397	0.1585	1.2462	0.033*	
C5	0.65016 (12)	0.3084 (2)	1.15104 (7)	0.0224 (3)	
H5A	0.6627	0.1916	1.1172	0.027*	
C6	0.72640 (11)	0.6918 (2)	0.95170 (6)	0.0183 (2)	
O1	0.8259 (9)	0.7831 (13)	0.9285 (10)	0.0169 (10)	0.589 (2)
C7	0.9256 (16)	0.674 (3)	0.9020 (10)	0.0235 (7)	0.589 (2)
H7A	0.8944	0.5470	0.8700	0.028*	0.589 (2)
H7B	0.9824	0.6178	0.9460	0.028*	0.589 (2)
C8	0.9949 (2)	0.8301 (4)	0.85523 (13)	0.0212 (3)	0.589 (2)
H8A	1.0637	0.7495	0.8364	0.025*	0.589 (2)
H8B	0.9383	0.8804	0.8101	0.025*	0.589 (2)
C9	1.0467 (2)	1.0298 (4)	0.90058 (13)	0.0232 (4)	0.589 (2)
H9A	0.9792	1.1045	0.9228	0.028*	0.589 (2)
H9B	1.1088	0.9809	0.9432	0.028*	0.589 (2)
C10	1.1062 (15)	1.191 (2)	0.8511 (9)	0.0263 (14)	0.589 (2)
H10A	1.1392	1.3158	0.8823	0.039*	0.589 (2)
H10B	1.0444	1.2426	0.8097	0.039*	0.589 (2)
H10C	1.1736	1.1174	0.8294	0.039*	0.589 (2)
O1B	0.8163 (14)	0.819 (2)	0.9292 (15)	0.0169 (10)	0.411 (2)
C7B	0.920 (2)	0.683 (4)	0.9022 (14)	0.0235 (7)	0.411 (2)
H7BA	0.9431	0.5589	0.9372	0.028*	0.411 (2)
H7BB	0.8949	0.6257	0.8500	0.028*	0.411 (2)
C8B	1.0250 (3)	0.8474 (5)	0.90349 (19)	0.0212 (3)	0.411 (2)
H8BA	1.0947	0.7751	0.8827	0.025*	0.411 (2)
H8BB	1.0537	0.8874	0.9573	0.025*	0.411 (2)
C9B	0.9920 (3)	1.0572 (6)	0.85830 (19)	0.0232 (4)	0.411 (2)
H9BA	0.9239	1.1321	0.8798	0.028*	0.411 (2)
H9BB	0.9617	1.0177	0.8047	0.028*	0.411 (2)
C10B	1.102 (2)	1.219 (3)	0.8590 (13)	0.0263 (14)	0.411 (2)
H10D	1.0730	1.3545	0.8324	0.039*	0.411 (2)
H10E	1.1663	1.1515	0.8331	0.039*	0.411 (2)
H10F	1.1351	1.2532	0.9119	0.039*	0.411 (2)
Mn1	0.5000	0.5000	1.0000	0.01767 (8)	
N1	0.58831 (10)	0.48802 (18)	1.12286 (6)	0.0197 (2)	
S1	0.61548 (3)	0.86094 (5)	0.978244 (17)	0.01905 (8)	
S2	0.71689 (3)	0.41428 (5)	0.953823 (19)	0.02316 (9)	

### Atomic displacement parameters (Å^2^)

**Table:**

	*U*^11^	*U*^22^	*U*^33^	*U*^12^	*U*^13^	*U*^23^
C1	0.0267 (6)	0.0216 (6)	0.0180 (6)	−0.0082 (5)	0.0053 (5)	−0.0013 (5)
C2	0.0331 (7)	0.0287 (7)	0.0171 (6)	−0.0124 (6)	0.0062 (5)	−0.0053 (5)
C3	0.0327 (7)	0.0350 (7)	0.0159 (6)	−0.0152 (6)	−0.0006 (5)	0.0009 (5)
C4	0.0289 (7)	0.0272 (7)	0.0236 (6)	−0.0073 (6)	−0.0031 (5)	0.0034 (5)
C5	0.0248 (6)	0.0224 (6)	0.0200 (6)	−0.0068 (5)	0.0025 (5)	−0.0012 (5)
C6	0.0209 (6)	0.0196 (6)	0.0137 (5)	−0.0060 (5)	−0.0001 (4)	0.0020 (4)
O1	0.0140 (14)	0.005 (3)	0.0317 (5)	0.0019 (17)	0.0048 (12)	0.006 (2)
C7	0.0200 (14)	0.0205 (13)	0.0304 (7)	−0.0033 (11)	0.0055 (8)	0.0032 (8)
C8	0.0172 (8)	0.0231 (8)	0.0237 (8)	−0.0035 (7)	0.0040 (7)	0.0021 (8)
C9	0.0226 (9)	0.0233 (8)	0.0232 (9)	−0.0059 (7)	0.0008 (6)	0.0035 (7)
C10	0.0269 (13)	0.022 (3)	0.031 (3)	−0.007 (2)	0.0053 (16)	0.003 (2)
O1B	0.0140 (14)	0.005 (3)	0.0317 (5)	0.0019 (17)	0.0048 (12)	0.006 (2)
C7B	0.0200 (14)	0.0205 (13)	0.0304 (7)	−0.0033 (11)	0.0055 (8)	0.0032 (8)
C8B	0.0172 (8)	0.0231 (8)	0.0237 (8)	−0.0035 (7)	0.0040 (7)	0.0021 (8)
C9B	0.0226 (9)	0.0233 (8)	0.0232 (9)	−0.0059 (7)	0.0008 (6)	0.0035 (7)
C10B	0.0269 (13)	0.022 (3)	0.031 (3)	−0.007 (2)	0.0053 (16)	0.003 (2)
Mn1	0.02238 (14)	0.01829 (14)	0.01273 (12)	−0.00792 (10)	0.00374 (9)	−0.00022 (9)
N1	0.0229 (5)	0.0207 (5)	0.0157 (5)	−0.0079 (4)	0.0037 (4)	−0.0008 (4)
S1	0.02207 (16)	0.01559 (14)	0.01983 (15)	−0.00504 (11)	0.00407 (11)	0.00058 (11)
S2	0.02738 (17)	0.01555 (15)	0.02887 (17)	−0.00708 (12)	0.01235 (13)	−0.00299 (12)

### Geometric parameters (Å, °)

**Table:**

C1—N1	1.3447 (17)	C9—H9B	0.9900
C1—C2	1.3937 (17)	C10—H10A	0.9800
C1—H1A	0.9500	C10—H10B	0.9800
C2—C3	1.384 (2)	C10—H10C	0.9800
C2—H2A	0.9500	O1B—C7B	1.52 (3)
C3—C4	1.387 (2)	C7B—C8B	1.52 (2)
C3—H3A	0.9500	C7B—H7BA	0.9900
C4—C5	1.3906 (18)	C7B—H7BB	0.9900
C4—H4A	0.9500	C8B—C9B	1.523 (5)
C5—N1	1.3454 (18)	C8B—H8BA	0.9900
C5—H5A	0.9500	C8B—H8BB	0.9900
C6—O1	1.332 (8)	C9B—C10B	1.55 (2)
C6—O1B	1.349 (11)	C9B—H9BA	0.9900
C6—S2	1.6925 (13)	C9B—H9BB	0.9900
C6—S1	1.7014 (13)	C10B—H10D	0.9800
O1—C7	1.41 (2)	C10B—H10E	0.9800
C7—C8	1.524 (16)	C10B—H10F	0.9800
C7—H7A	0.9900	Mn1—N1^i^	2.2558 (11)
C7—H7B	0.9900	Mn1—N1	2.2558 (11)
C8—C9	1.523 (3)	Mn1—S1	2.5863 (4)
C8—H8A	0.9900	Mn1—S1^i^	2.5863 (4)
C8—H8B	0.9900	Mn1—S2	2.6554 (5)
C9—C10	1.513 (16)	Mn1—S2^i^	2.6554 (5)
C9—H9A	0.9900		
			
N1—C1—C2	122.57 (13)	O1B—C7B—H7BA	111.1
N1—C1—H1A	118.7	C8B—C7B—H7BB	111.1
C2—C1—H1A	118.7	O1B—C7B—H7BB	111.1
C3—C2—C1	118.86 (13)	H7BA—C7B—H7BB	109.1
C3—C2—H2A	120.6	C7B—C8B—C9B	114.6 (9)
C1—C2—H2A	120.6	C7B—C8B—H8BA	108.6
C2—C3—C4	118.85 (12)	C9B—C8B—H8BA	108.6
C2—C3—H3A	120.6	C7B—C8B—H8BB	108.6
C4—C3—H3A	120.6	C9B—C8B—H8BB	108.6
C3—C4—C5	119.09 (14)	H8BA—C8B—H8BB	107.6
C3—C4—H4A	120.5	C8B—C9B—C10B	113.5 (9)
C5—C4—H4A	120.5	C8B—C9B—H9BA	108.9
N1—C5—C4	122.39 (13)	C10B—C9B—H9BA	108.9
N1—C5—H5A	118.8	C8B—C9B—H9BB	108.9
C4—C5—H5A	118.8	C10B—C9B—H9BB	108.9
O1—C6—S2	118.6 (4)	H9BA—C9B—H9BB	107.7
O1B—C6—S2	128.8 (6)	C9B—C10B—H10D	109.5
O1—C6—S1	118.1 (4)	C9B—C10B—H10E	109.5
O1B—C6—S1	107.9 (6)	H10D—C10B—H10E	109.5
S2—C6—S1	123.34 (7)	C9B—C10B—H10F	109.5
C6—O1—C7	127.1 (9)	H10D—C10B—H10F	109.5
O1—C7—C8	110.3 (11)	H10E—C10B—H10F	109.5
O1—C7—H7A	109.6	N1^i^—Mn1—N1	180.0
C8—C7—H7A	109.6	N1^i^—Mn1—S1	89.15 (3)
O1—C7—H7B	109.6	N1—Mn1—S1	90.85 (3)
C8—C7—H7B	109.6	N1^i^—Mn1—S1^i^	90.86 (3)
H7A—C7—H7B	108.1	N1—Mn1—S1^i^	89.15 (3)
C9—C8—C7	112.9 (7)	S1—Mn1—S1^i^	180.0
C9—C8—H8A	109.0	N1^i^—Mn1—S2	89.85 (3)
C7—C8—H8A	109.0	N1—Mn1—S2	90.15 (3)
C9—C8—H8B	109.0	S1—Mn1—S2	69.478 (12)
C7—C8—H8B	109.0	S1^i^—Mn1—S2	110.523 (12)
H8A—C8—H8B	107.8	N1^i^—Mn1—S2^i^	90.16 (3)
C10—C9—C8	111.8 (5)	N1—Mn1—S2^i^	89.84 (3)
C10—C9—H9A	109.3	S1—Mn1—S2^i^	110.521 (12)
C8—C9—H9A	109.3	S1^i^—Mn1—S2^i^	69.477 (12)
C10—C9—H9B	109.3	S2—Mn1—S2^i^	180.0
C8—C9—H9B	109.3	C1—N1—C5	118.23 (11)
H9A—C9—H9B	107.9	C1—N1—Mn1	120.78 (9)
C6—O1B—C7B	112.4 (12)	C5—N1—Mn1	120.81 (8)
C8B—C7B—O1B	103.3 (16)	C6—S1—Mn1	84.58 (4)
C8B—C7B—H7BA	111.1	C6—S2—Mn1	82.56 (4)
			
N1—C1—C2—C3	0.2 (2)	S1^i^—Mn1—N1—C1	−121.11 (9)
C1—C2—C3—C4	0.7 (2)	S2—Mn1—N1—C1	128.36 (9)
C2—C3—C4—C5	−0.8 (2)	S2^i^—Mn1—N1—C1	−51.64 (9)
C3—C4—C5—N1	0.0 (2)	S1—Mn1—N1—C5	−126.13 (9)
O1B—C6—O1—C7	−171 (12)	S1^i^—Mn1—N1—C5	53.87 (9)
S2—C6—O1—C7	3(2)	S2—Mn1—N1—C5	−56.65 (9)
S1—C6—O1—C7	−177.8 (13)	S2^i^—Mn1—N1—C5	123.35 (9)
C6—O1—C7—C8	159.6 (13)	O1—C6—S1—Mn1	178.4 (9)
O1—C7—C8—C9	59.6 (13)	O1B—C6—S1—Mn1	177.2 (12)
C7—C8—C9—C10	−175.5 (10)	S2—C6—S1—Mn1	−1.95 (7)
O1—C6—O1B—C7B	7(9)	N1^i^—Mn1—S1—C6	−89.05 (5)
S2—C6—O1B—C7B	0(3)	N1—Mn1—S1—C6	90.95 (5)
S1—C6—O1B—C7B	−178.7 (15)	S2—Mn1—S1—C6	1.11 (4)
C6—O1B—C7B—C8B	−163.2 (16)	S2^i^—Mn1—S1—C6	−178.89 (4)
O1B—C7B—C8B—C9B	−54.0 (18)	O1—C6—S2—Mn1	−178.5 (9)
C7B—C8B—C9B—C10B	−178.7 (15)	O1B—C6—S2—Mn1	−177.1 (14)
C2—C1—N1—C5	−0.99 (19)	S1—C6—S2—Mn1	1.91 (7)
C2—C1—N1—Mn1	174.12 (10)	N1^i^—Mn1—S2—C6	88.03 (5)
C4—C5—N1—C1	0.90 (19)	N1—Mn1—S2—C6	−91.97 (5)
C4—C5—N1—Mn1	−174.21 (10)	S1—Mn1—S2—C6	−1.12 (4)
S1—Mn1—N1—C1	58.88 (9)	S1^i^—Mn1—S2—C6	178.88 (4)

Symmetry codes: (i) −*x*+1, −*y*+1, −*z*+2.
